# Erdheim-Chester disease: a rare histiocytosis (case report and review of the literature)

**DOI:** 10.11604/pamj.2018.29.62.4088

**Published:** 2018-01-22

**Authors:** Mahtat El Mehdi, Safae Regragui, Hicham Eddou, Selim Jennane, Hicham El Maaroufi, Kamal Doghmi, Mohamed Mikdame

**Affiliations:** 1 Department of Clinical Haematology, Military Hospital of Instruction Mohamed V, Rabat, Morocco

**Keywords:** Erdheim-Chester disease, polydipsia-polyuria syndrome, MRI

## Abstract

We report a case of Erdheim-chester disease, a rare non-langerhans histiocytosis revealed by polydipsia-polyuria syndrome in a 26 years old woman, trated by interferon with a good response.

## Introduction

Erdheim-Chester disease (ECD) is a rare, non-inherited, non-Langerhans form of histiocytosis of unknown origin. [1] First described in 1930 by William Chester as “lipoid granulomatose” [2], ECD is characterized by tissue xanthomatous infiltration by spumous histiocytes. Immunohistochemical staining results are positive for CD68 and negative for CD1a, whereas those for S-100 protein may be negative or positive. [3] The treatment is essentially based on interferon alpha administration. We report in this paper, a typical ECD in a young woman revealed by polydipsia-polyuria syndrome secondary to a hypophyseal infiltration, with good response to treatment by interferon alpha.

## Patient and observation

**Figure 1:**
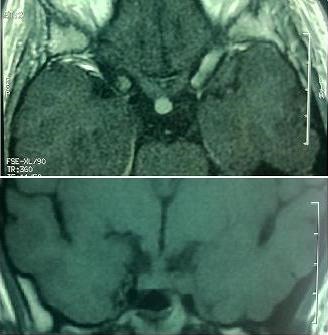
Hypothalamo-pituitary MRI showing a thickened pituitary stalk (7mm) with gadolinium enhancement and lost of the hyperintense signal on T1-weighed image

**Figure 2:**
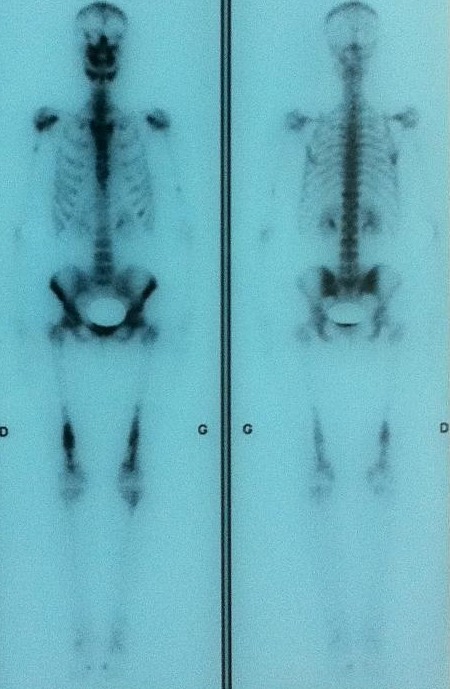
A technetium-99m-methyl-diphosphonate skeletal scintigraphy showing diffuse uptake in the diaphysis of long bones

**Figure 3:**
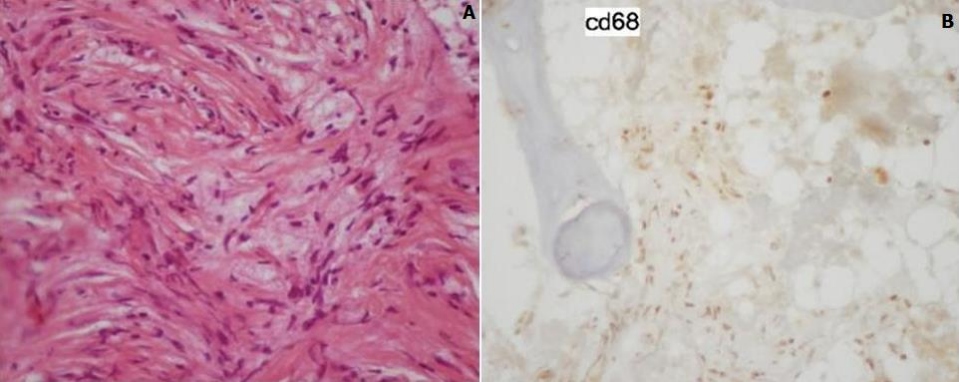
A) inflammatory infiltrate with lymphocytes and spumous macrophages; B) positive CD68 foamy histiocytes

A 26 years old woman from Morocco presented with a 2-months history of polydipsia-polyuria, headache and mild blurred vision. The patient reported also a mild pain in both inferior limbs. The physical examination was normal. Ocular fundus exam showed a bilateral mild papillary edema. Serum sodium was normal and fluid deprivation test resulted in a severe dehydration (the patient lost 5% of her weight) without urine increased concentration. The desmopressin (DDAVP) stimulation test was positive. Pituitary hormones dosing was normal. Other Biologic investigation showed a mild inflammatory syndrome with a C-reactive protein at 21,1 mg/L ( N < 3 mg/L) and a polyclonal hyper-gammaglobulinemia at 17g/L (N: 8-13,5 g/L). Hypothalamo-pituitary MRI showed a thickened pituitary stalk (7 mm) with gadolinium enhancement and lost of the hyperintense signal on T1-weighed image (Figure 1). We completed the work up with a technetium-99m-methyl-diphosphonate skeletal scintigraphy which showed diffuse uptake of the radiopharmaceutical, in the diaphysis of long bones (Figure 2). Computed tomography scan-guided femoral bone biopsies showed an inflammatory infiltrate with lymphocytes and spumous macrophages (Figure 3). Immunohistochemically, foamy histiocytes were positive for CD68 and negative for the S-100 protein, CD1a (Figure 3). Echocardiography was normal. The patient was treated by Interferon-alpha (9 mIU/week) associated to nasal DDAVP. The treatment was well tolerated. After 6 months, the symptoms disappeared. The subsequent MRI showed an improvement with a decrease of the stalk thickening and contrast enhancement.

## Discussion

Erdheim-Chester is a rare non-Langerhans histiocytosis. Its pathogeny remains unclear. A recent review indicates 445 published cases in the literature [4]. There are no diagnostic criteria for this entity, and diagnosis is usually based on radiologic findings of osteosclerosis combined with histopathological evidence of foamy CD68 positive and CD1a negative histiocytic infiltration [5]. ECD patients present with heterogenous clinical manifestations. Bone pain is the most common symptom in those patients. Symmetrical radiodensities in the metaphyseal and diaphyseal portions of long bones, and abnormally increased labeling in the long bones of the lower limbs on technetium-99 bone scans as found in our patient, are typical hallmarks [1]. Half of the ECD patients have extra-skeletal manifestations; the most common are exophthalmos, diabetes insipidus and skin involvement. Some patients may have general symptoms (fever, weight loss) [5]. Central nervous system involvement is found in about half of the patients, it includes diencephalic, meningeal, cerebellar infiltration [5]. Hypophyseal infiltration, which causes diabetes insipidus, is defined as the presence of a pituitary infiltration on brain MRI and/or CT scan or as the lack of the normal T1 high signal intensity of the posterior pituitary lobe MRI as reported in our case [6]. Cardiovascular infiltration is reported in 40% of ECD cases. Periaortic fibrosis is the most frequent vascular involvement with sometimes a “coated aorta” aspect. Other arteries infiltration are described in this disease like renal artery stenosis or life threatening ischemic manifestations like cerebral ischemia, myocardial infarction or mesenteric angina. Some patients may present with pericarditis possibly leading to tamponade [1]. The prognosis of this histiocytosis is relatively poor, as reported in Veyssier-Belot et *al review*, 22 (59%) of 37 patients died in a mean follow up of 2.7 years. Common causes of death were cadiac failure and pulmonary fibrosis [5]. The first line treatment of ECD is based on interferon-α administration which improves survival in multisystemic disease, it should be prescribed at high dose if there is central nervous system and/or cardiovascular involvement [7]. Indeed, we have now treated our patient with interferon alpha for 12 months without serious complications. Although the clinical symptoms of our patient seem to be stable, the efficacy of interferon alpha has been reported to be limited especially in CNS and cardiovascular involvement [1, 7]. The optimal duration of interferon remains unknown. Other effective treatments have been reported being effective in some case reports or small series, such as steroids, biphosphonates, imatinib mesylate, cladribine and autologous stem cell transplantation. Recently, it was found that BRAF V600E mutation is recurrent in this disease; about half of ECD patients carry this mutation [8]. Thus Vemurafenib, an inhibitor of mutant BRAF, might be an interesting effective treatment alternative of the ECD as reported recently in three multisystemic and refractory ECD patients [9].

## Conclusion

ECD is a very rare histiocytosis as about 450 cases have been reported till now. Its incidence might be underestimated due to lack of knowledge and difficulty of diagnosis. Although its prognosis remains poor, the recent discovery of the recurrent BRAF mutation and the spectacular response to BRAF targeted therapy might improve the outcome of ECD patients.

## Competing interests

The authors declare no competing interest.
